# Top-down Effects on Empathy for Pain in Adults with Autistic Traits

**DOI:** 10.1038/s41598-019-44400-2

**Published:** 2019-05-29

**Authors:** Jing Meng, Lin Shen, Zuoshan Li, Weiwei Peng

**Affiliations:** 1 Key Laboratory of Applied Psychology, Chongqing Normal University, Chongqing, China; 2 School of Education, Chongqing Normal University, Chongqing, China; 3 School of Mathematical Sciences, Chongqing Normal University, Chongqing, China; 4 College of Psychology, Shenzhen University, Shenzhen, China; 5 Shenzhen Key Laboratory of Affective and Social Cognitive Science, Shenzhen University, Shenzhen, China

**Keywords:** Emotion, Human behaviour

## Abstract

While empathic responses of individuals with autism-spectrum disorder have been reported to be modulated by top-down attention, it remains unclear whether empathy for pain in typically developing individuals with autistic traits also involves such top-down modulation mechanisms. This study employed the autism-spectrum quotient (AQ) to quantify autistic traits in a group of 1,231 healthy adults. Two subset groups (High-AQ and Low-AQ groups) were randomly selected from the highest and lowest 10% AQ scores respectively. We explored whether participants in both groups would differ in their response to others’ pain when their attention was directed toward (A-P tasks) or away (A-N tasks) from pain cues in auditory and visual experimental modalities. Compared to Low-AQ individuals, High-AQ individuals exhibited more suppressed N1 and P2 amplitudes in response to painful vocal cues in auditory A-N tasks. This suggests suppressed attentional and emotional processes of empathy for pain when High-AQ individuals have their attention directed away from others’ pain cues. No significant difference was found between both groups in the auditory A-P task, nor in the visual A-P and A-N tasks. These results suggest that top-down attention modulation of cortical empathic responses to others’ vocal pain is influenced by autistic traits.

## Introduction

As a social species, typically developing (TD) human individuals have the ability to empathize with others, i.e., comprehend others’ emotions and feelings as if these were their own^1,2^. By extension, empathy for pain implies the ability to perceive and judge the pain of others^1^. Indeed, empathy for pain enables TD individuals to understand the subjective painful experience of another person, which subsequently influences the regulation of appropriate social interactions.

The modulation of top-down attention on the empathic processing of pain in the visual modality has been well documented^3,4^. The attention allocation of participants is typically manipulated via two different task instructions: (1) a pain judgement task, where participants are instructed to judge the pain experienced by models depicted in pictures, which usually requires participants to direct their attention to the pain cues; (2) a number-counting task, where participants are instructed to count the number of hands depicted in pictures, which directs participants’ attention away from the models’ feelings. In contrast to the number-counting task, the pain judgement task results in increased activation of the pain matrix (e.g., insula, paracingulate, and the left middle frontal gyrus)^4^ and a positive shift at late event-related potential (ERP) components (P3 and LPC), indexing the cognitive evaluation of others’ pain^3^ in response to the presented pictures. These results indicate that the process of evaluation and judgement of others’ pain is influenced by the attention allocation to the depicted pain cues. Moreover, amplitudes of early ERP components to empathy for pain (N1 and P2) over the frontal-central area were correlated with subjective reports of the degree of perceived pain in others and of the participants’ personal discomfort. These results indicate that the early automatic response indexes emotional sharing and the late response indexes the cognitive evaluation of others’ pain^3^.

Autism spectrum disorder (ASD) is characterized by persistent deficits in communication and social interactions; individuals with ASD display restricted and repetitive patterns of behaviour, interests, or activities^5^. The autism-spectrum quotient (AQ)^6^ has been widely adopted to evaluate individual autistic traits. Individuals with ASD scored at the extreme end of the AQ distribution across the entire population^7^. Based on this, it has been reported that individuals with ASD and TD groups with autistic traits at a subclinical level share similar behavioural patterns^8,9^. A number of studies suggested that empathic impairment to be involved in ASD individuals^6^ and TD groups with autistic traits^10,11^; however, relevant evidence for such a deficit is varied and subtle^12^.

The effects of top-down attention modulation on empathic abilities have been studied in individuals with ASD^12^. In particular, without directly asking individuals who are involved in an explicit empathic task for a verbal report, Kliemann *et al*. proposed that implicit empathic processes involved a more indirect assessment^13^. Thus, the authors used both explicit and implicit tasks to investigate facial emotional recognition processes in both an ASD group and a control group. In the explicit task, participants were instructed to focus their attention to the emotional aspects of the provided stimuli (i.e., label an emotional expression). In the implicit task, participants were instructed to direct their attention away from the emotional aspects of the stimuli (i.e., watch a video that shows eyes and locate the video that shows the corresponding mouth). The between-group difference was larger for the implicit task compared to the explicit task, thus suggesting increased implicit processing impairments for facial expression recognition in ASD individuals^13^. Explicit empathy is typically considered as conscious and controlled processing, while implicit empathy is typically considered to be a more automatic occurrence outside of the conscious awareness^14^. Furthermore, it has been suggested that top-down attention may play an important role in ASD individuals’ explicit and implicit empathic responses to others’ expressions^13^. If participants do not pay explicit attention to others’ feelings, their empathic responses would be impaired. Since the processes of empathy for pain may not be identical to the recognition of facial expressions, it remains an open question whether empathy for pain in individuals with both ASD and autistic traits would be modulated by top-down attention.

Here, the influence of autistic traits on the top-down attention modulation of empathy for pain was tested to achieve a better understanding of the cognitive and neural mechanisms underlying empathic deficits in individuals with autistic traits. In particular, an empathy for pain task in both visual and auditory modalities was adopted, assuming the recognition of pain from vocal cues of others as the auditory equivalent of visual injury recognition. Building upon previous studies^3,4^, the participants’ attention regarding the pain cues was manipulated by either instructing them to judge whether the stimuli were painful or non-painful, or, in contrast, to count the number of hands visible in a given picture (in the visual experiment), or to judge the speakers’ gender (in the auditory experiment). The temporal dynamics of the neural mechanisms underlying empathy for pain were explored by recording ERPs elicited in both TD individuals with high AQ scores (High-AQ) and in TD individuals with low AQ scores (Low-AQ) by viewing pictures and hearing the voices of people in pain. Furthermore, the top-down attention modulation of empathic responses was compared between Low-AQ and High-AQ participants in both visual and auditory modalities.

Based on evidence indicating that ASD individuals have impaired implicit processing in empathic processing^8,13^, we hypothesized that High-AQ individuals would show a decreased implicit response to others’ pain compared to to Low-AQ individuals. More specifically, High-AQ individuals would stronger suppress ERP responses to others’ pain when their attention is directed away from pain cues than Low-AQ individuals. Since the differences of behavioural responses to empathy for pain between High-AQ and Low-AQ individuals were selectively observed in the auditory modality^15^, we hypothesized that between-group differences of ERP responses to empathy for pain would also be present in the auditory modality.

## Results

### Behavioural data

Accuracies (ACCs), reaction times (RTs), and inverse efficiency scores (IESs) were compared via four-way repeated-measures analyses of variance (ANOVA), using the three within-participants factors of “modality” (visual vs. auditory), “pain” (painful vs. non-painful), and “task” (A-P vs. A-N), as well as the between-participants factor of “group” (High-AQ vs. Low-AQ). Mean ACCs, RTs, and IESs in response to both painful and non-painful stimuli in all conditions are summarized in Table 1. A full list of all statistical comparisons can be found in Table 2.

**Table 1:** Results of behavioural data.

Experiment	Group	ACC (%)				RT (ms)				Pain intensity ratings		Emotional reactions	
		Painful		Non-painful		Painful		Non-painful		Painful	Non- painful	Painful	Non- painful
		A-P	A-N	A-P	A-N	A-P	A-N	A-P	A-N				
Visual	High-AQ	95.6 (2.1)	97.5 (1.8)	93.9 (7.4)	95.5 (2.2)	832.36 (147.33)	701.31 (99.42)	891.16 (141.09)	659.98 (77.59)	6.14 (0.67)	1.48 (0.63)	6.56 (0.63)	4.61 (0.71)
	Low-AQ	92.5 (4.5)	97.8 (2.9)	95.0 (2.8)	95.7 (1.3)	790.16 (152.59)	671.90 (104.74)	807.23 (148.30)	623.05 (91.56)	6.17 (0.99)	1.71 (0.59)	4.28 (1.06)	6.64 (1.16)
Auditory	High-AQ	88.2 (13.5)	98.2 (2.6)	95.6 (5.1)	76.3 (18.2)	910.88 (210.92)	734.72 (134.36)	906.52 (182.56)	818.84 (181.39)	5.92 (1.15)	1.67 (0.63)	6.12 (0.96)	4.32 (1.00)
	Low-AQ	95.5 (3.9)	97.3 (4.4)	94.3 (5.9)	76.9 (15.0)	692.62 (201.52)	638.55 (164.64)	711.85 (199.56)	708.59 (202.53)	6.32 (1.47)	1.51 (0.43)	6.49 (1.36)	4.72 (0.80)

Note. Mean response ACCs (%), RTs (ms), pain intensity ratings, and emotional reactions (Mean (Standard deviation)) in each condition were present in the table.

**Table 2:** Summary of statistical analysis of behavioural data.

Experiment	ACC			RT			IES			Pain intensity ratings			Emotional reactions		
	*F*	*p*	*η* ^2^	*F*	*p*	*η* ^2^	*F*	*p*	*η* ^2^	*F*	*p*	*η* ^2^	*F*	*p*	*η* ^2^
Task	**4**.**766**	**0**.**038**	**0**.**145**	**56**.**524**	<**0**.**001**	**0**.**669**	**18**.**712**	<**0**.**001**	**0**.**401**						
Pain	**25**.**599**	<**0**.**001**	**0**.**478**	**9**.**516**	**0**.**005**	**0**.**254**	**16**.**692**	<**0**.**001**	**0**.**373**	**670**.**056**	<**0**.**001**	**0**.**960**	**72**.**293**	<**0**.**001**	**0**.**721**
Group	0.229	0.636	0.008	**4**.**726**	**0**.**038**	**0**.**144**	**4**.**625**	**0**.**040**	**0**.**142**	0.435	0.515	0.015	0.441	0.512	0.016
Modality	**22**.**647**	<**0**.**001**	**0**.**447**	0.681	0.416	**0**.**024**	**8**.**580**	**0**.**007**	**0**.**235**	0.016	0.899	0.001	1.089	0.306	0.037
Task × Group	0.291	0.594	0.010	4.124	0.052	0.128	4.005	0.055	0.125						
Pain × Group	0.154	0.697	0.005	0.628	0.435	0.022	0.051	0.822	0.002	0.269	0.608	0.010	0.172	0.681	0.006
Modality × Group	0.703	0.409	0.025	**5**.**869**	**0**.**022**	**0**.**173**	**4**.**993**	**0**.**034**	**0**.**151**						
Task × Pain	**41**.**156**	<**0**.**001**	**0**.**595**	0.254	0.618	0.009	**14**.**232**	**0**.**001**	**0**.**337**						
Modality × Task	**26**.**527**	<**0**.**001**	**0**.**486**	**10**.**874**	**0**.**003**	**0**.**280**	**23**.**963**	<**0**.**001**	**0**.**461**						
Modality × Task × Group	1.711	0.201	0.058	1.984	0.170	0.066	3.163	0.086	0.102						
Modality × Pain × Group	2.095	0.159	0.070	0.627	0.435	0.022	1.146	0.294	0.039	2.280	0.142	0.075	0.435	0.515	0.015
Pain × Group × Task	0.475	0.496	0.017	0.004	0.949	0.001	0.841	0.367	0.029						
Modality × Task × Pain	**28**.**547**	<**0**.**001**	**0**.**505**	**25**.**492**	<**0**.**001**	**0**.**477**	**28**.**853**	<**0**.**001**	**0**.**507**						
Modality × Pain × Group × Task	3.116	0.088	0.100	1.413	0.244	0.048	2.579	0.119	0.084						

Note: df:(1,28) The significant (*p* < 0.05) comparisons were shown in boldface.

RTs and IESs were significantly modulated by “group” [RTs: *F*(1,28) = 4.726, *p* = 0.038, *η*^2^ = 0.144; IESs: *F*(1,28) = 4.625, *p* = 0.040, *η*^2^ = 0.142], indicating that the High-AQ group responded more slowly than the Low-AQ group. RTs and IESs were also significantly modulated by the interaction between “group” and “modality” [RTs: *F*(1,28) = 5.869, *p* = 0.022, *η*^2^ = 0.173; IESs: *F*(1,28) = 4.993, *p* = 0.034, *η*^2^ = 0.151]. Post hoc ANOVA indicated that the High-AQ group was slower than the Low-AQ group in the auditory modality [RTs: *F*(1,28) = 6.489, *p* = 0.017, *η*^2^ = 0.188; IESs: *F*(1,28) = 5.604, *p* = 0.025, *η*^2^ = 0.167]. However, no significant difference was found in the visual modality [RTs: *F*(1,28) = 1.417, *p* = 0.244, *η*^2^ = 0.048; IESs: *F*(1,28) = 1.450, *p* = 0.239, *η*^2^ = 0.049].

ACCs and IESs were significantly modulated by “modality” [ACCs: *F*(1,28) = 22.647, *p* < 0.001, *η*^2^ = 0.447; IESs: *F*(1,28) = 8.580, *p* = 0.007, *η*^2^ = 0.235], indicating that the participants judged visual stimuli more accurately than auditory stimuli. In addition, ACCs and IESs displayed significant interaction effects between “task” and “pain” [ACCs: *F*(1,28) = 41.156, *p* < 0.001, *η*^2^ = 0.595; IESs: *F*(1,28) = 14.232, *p* = 0.001, *η*^2^ = 0.337]. Participants judged painful stimuli more accurately than non-painful stimuli in the A-N task [ACCs: *F*(1,28) = 55.576 *p* < 0.001, *η*^2^ = 0.665; IESs: *F*(1,28) = 35.135, *p* < 0.001, *η*^2^ = 0.557]. However, no significant difference was found between painful and non-painful stimuli in the A-P task [ACCs: *F*(1,28) = 1.823, *p* = 0.188, *η*^2^ = 0.061; IESs: *F*(1,28) = 0.014, *p* = 0.908, *η*^2^ < 0.001].

ACCs, RTs, and IESs were all significantly modulated by “task” [ACCs: *F*(1,28) = 4.766, *p* = 0.038, *η*^2^ = 0.145; RTs: *F*(1,28) = 56.524, *p* < 0.001, *η*^2^ = 0.669; IESs: *F*(1,28) = 18.712, *p* < 0.001, *η*^2^ = 0.401] and by “pain” [ACCs: *F*(1,28) = 25.599, *p* < 0.001, *η*^2^ = 0.478; RTs: *F*(1,28) = 9.516, *p* = 0.005, *η*^2^ = 0.254; IESs: *F*(1,28) = 16.692, *p* < 0.001, *η*^2^ = 0.373]. Participants responded significantly more accurately but slower to the A-P task than to the A-N task; participants responded significantly more accurately and faster to the painful stimuli than to the non-painful stimuli. In addition, ACCs, RTs, and IESs displayed significant interaction effects between “modality” and “task” [ACCs: *F*(1,28) = 26.527, *p* < 0.001, *η*^2^ = 0.486; RTs: *F*(1,28) = 10.874, *p* = 0.003, *η*^2^ = 0.280; IESs: *F*(1,28) = 23.963, *p* < 0.001, *η*^2^ = 0.461], as well as between “modality”, “task”, and “pain” [ACCs: *F*(1,28) = 28.547, *p* < 0.001, *η*^2^ = 0.505; RTs: *F*(1,28) = 25.492, *p* < 0.001, *η*^2^ = 0.477; IESs: *F*(1,28) = 28.853, *p* < 0.001, *η*^2^ = 0.507]. ACCs, RTs, and IESs showed significant differences between visual and auditory modalities for non-painful stimuli in A-N tasks (all *p* values < 0.001). Participants judged more accurately and faster in the visual modality than in the auditory modality, whereas no significant difference was found between visual and auditory modalities in other conditions (all *p* values > 0.05).

### ERP data

Amplitudes and latencies of ERP components were compared via mixed model ANOVA using the between-participants factor “group” and the within-participants factors “task” and “pain”.

### ERP data from the visual experiment

**Figure 1:**
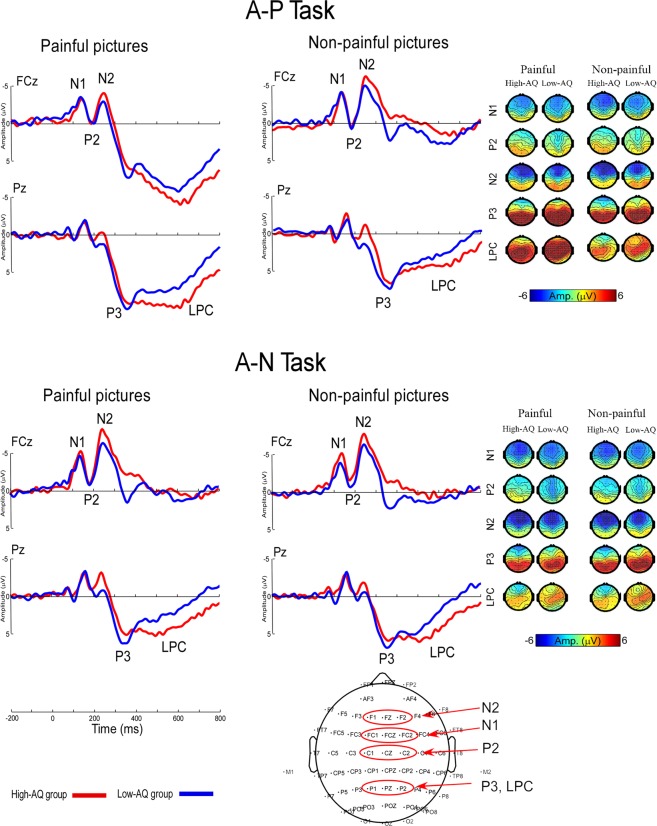
Cortical responses to painful and non-painful pictures at FCz and Pz in the visual experiment. Top panel: ERP waves and voltage scalp maps of the High-AQ and Low-AQ group in response to the painful and non-painful pictures in the Visual A-P task. Middle panel: ERP waves and voltage scalp maps of the High-AQ and Low-AQ group in response to the painful and non-painful pictures in the Visual A-N task. Red lines illustrate averaged amplitudes of the High-AQ group. Blue lines illustrate averaged amplitudes of the Low-AQ group. No difference was found between the two groups in response to painful and non-painful pictures in both of the A-P and A-N tasks (*p* > 0.05). Topographic plots at the bottom illustrate the electrodes considered in the analysis.

Averaged ERP waveforms and scalp topographies related to both painful and non-painful pictures in each condition are shown in Figure 1. A full list of all statistical comparisons can be found in Table 3.

**Table 3:** Summary of statistical analysis of ERP data in the visual experiment.

		N1			P2			N2			P3			LPC		
		*F*	*p*	*η* ^2^	*F*	*p*	*η* ^2^	*F*	*p*	*η* ^2^	*F*	*p*	*η* ^2^	*F*	*p*	*η* ^2^
Task	**amplitude**	**4.597**	**0.041**	**0.141**	**13.498**	**<0.001**	**0.325**	**6.791**	**0.015**	**0.195**	**37.636**	**<0.001**	**0.573**	**39.123**	**<0.001**	**0.583**
	**latency**	0.184	0.671	0.007	0.047	0.830	0.002	0.011	0.918	0.000	0.220	0.643	0.008			
Pain	**amplitude**	0.041	0.841	0.001	1.762	0.195	0.059	0.881	0.356	0.031	**9.536**	**0.005**	**0.254**	**53.774**	**<0.001**	**0.658**
	**latency**	0.374	0.546	0.013	0.018	0.894	0.001	1.983	0.170	0.066	0.060	0.809	0.002			
Group	**amplitude**	0.351	0.558	0.012	2.803	0.105	0.091	1.175	0.288	0.040	0.232	0.634	0.008	1.429	0.242	0.049
	**latency**	0.330	0.570	0.012	0.786	0.383	0.027	0.310	0.582	0.011	**12.072**	**0.002**	**0.301**			
Task × Group	**amplitude**	0.012	0.912	0.000	0.034	0.855	0.001	0.240	0.628	0.008	0.067	0.798	0.002	0.131	0.720	0.005
	**latency**	0.014	0.908	0.000	0.005	0.943	0.000	0.901	0.351	0.031	0.605	0.443	0.021			
Pain × Group	**amplitude**	**5.249**	**0.030**	**0.158**	1.906	0.178	0.064	2.634	0.116	0.086	**6.491**	**0.017**	**0.188**	1.350	0.255	0.046
	**latency**	0.001	0.977	0.000	0.292	0.593	0.010	0.088	0.769	0.003	0.477	0.496	0.017			
Task × Pain	**amplitude**	1.446	0.239	0.049	0.480	0.494	0.017	3.153	0.087	0.101	**38.067**	**<0.001**	**0.576**	**57.614**	**<0.001**	**0.673**
	**latency**	0.343	0.563	0.012	0.062	0.806	0.002	**5.663**	**0.024**	**0.168**	3.768	0.062	0.119			
Task × Pain × Group	**amplitude**	0.109	0.744	0.004	0.582	0.452	0.020	2.326	0.138	0.077	3.413	0.075	0.109	0.459	0.504	0.016
	**latency**	0.594	0.447	0.021	0.298	0.590	0.011	0.254	0.618	0.009	1.192	0.284	0.041			

Note: df:(1,28). The significant comparisons (*p* < 0.05) were shown in boldface.

#### ERPs amplitude

N1, P2, N2, P3, and LPC amplitudes elicited by visual stimuli were modulated by “task” [N1: *F*(1,28) = 4.597, *p* = 0.041, *η*^2^ = 0.141; P2: *F*(1,28) = 13.498, *p* < 0.001, *η*^2^ = 0.325; N2: *F*(1,28) = 6.791, *p* = 0.015, *η*^2^ = 0.195; P3: *F*(1, 28) = 37.636, *p* < 0.001, *η*^2^ = 0.573; LPC: *F*(1,28) = 39.123, *p* < 0.001, *η*^2^ = 0.583]. The N1 and N2 amplitudes were larger in the A-N task (N1: −6.27 ± 0.45 μV; N2: −7.29 ± 0.67 μV) than in the A-P task (N1: −5.70 ± 0.49 μV; N2: −6.25 ± 0.73 μV); the P2, P3, and LPC amplitudes were smaller in the A-N task (P2: 0.60 ± 0.55 μV; P3: 7.83 ± 1.01 μV; LPC: 3.04 ± 0.76 μV) than in the A-P task (P2: 1.74 ± 0.60 μV; P3: 10.04 ± 1.11 μV; LPC: 5.84 ± 0.92 μV), indicating a more negative deflection of ERP waves in the A-N task than in the A-P task.

The P3 and LPC amplitudes were significantly modulated by “pain” [P3: *F*(1,28) = 9.536, *p* = 0.005, *η*^2^ = 0.254; LPC: *F*(1, 28) = 53.774, *p* < 0.001, *η*^2^ = 0.658] and the interaction between “task” and “pain” [P3: *F*(1,28) = 38.067, *p* < 0.001, *η*^2^ = 0.576; LPC: *F*(1, 28) = 57.614, *p* < 0.001, *η*^2^ = 0.673]. Pictures containing pain cues elicited higher amplitudes (P3: 9.30 ± 1.08 μV; LPC: 5.29 ± 0.83 μV) than pictures that did not (P3: 8.57 ± 1.03 μV; LPC: 3.59 ± 0.82 μV). Post hoc two-way ANOVA of “task” and “pain” showed that larger amplitudes for painful pictures were elicited in the A-P task (P3: 11.24 ± 1.19 μV; LPC: 7.78 ± 0.98 μV) and not in the A-N task (P3: 7.35 ± 1.01 μV; LPC: 2.80 ± 0.76 μV) [P3: *F*(1,28) = 78.201, *p* < 0.001, *η*^2^ = 0.736; LPC: *F*(1, 28) = 83.284, *p* < 0.001, *η*^2^ = 0.748], while amplitudes for non-painful pictures did neither differ in the A-P task (P3: 8.84 ± 1.06 μV; LPC: 3.90 ± 0.93 μV) nor in the A-N task (P3: 8.30 ± 1.05 μV; LPC: 3.28 ± 0.78 μV) [P3: *F*(1,28) = 1.391, *p* = 0.248, *η*^2^ = 0.047; LPC: *F*(1,28) = 0.247, *p* < 0.001, *η*^2^ = 0.048].

#### ERPs latency

The P3 latencies were significantly modulated by “group” [*F*(1,28) = 6.033, *p* = 0.021, *η*^2^ = 0.177], where the High-AQ group (384.70 ± 7.07 ms) showed longer peak latencies than the Low-AQ group (349.97 ± 7.07 ms). The N2 latencies were modulated by the interaction between “task” and “pain” [*F*(1,28) = 38.067, *p* < 0.001, *η*^2^ = 0.576]. Post hoc two-way ANOVA showed that latencies of painful pictures (250.93 ± 4.18 ms) were shorter than those of non-painful pictures (267.33 ± 6.90 ms) in the A-P task [*F*(1,28) = 5.878, *p* = 0.022, *η*^2^ = 0.173], while latencies of painful (260.80 ± 8.00 ms) and non-painful pictures (258.93 ± 7.22 ms) did not differ in the A-N task [*F*(1,28) = 0.094, *p* = 0.761, *η*^2^ = 0.003].

#### Subjective reports and their correlation with neural activity

All mean scores and standard deviations of subjective reports are listed in Table 1. Participants judged painful pictures with higher pain intensities [*F*(1,28) = 936.085, *p* < 0.001, *η*^2^ = 0.971] and more negative emotional reactions [*F*(1,28) = 77.552, *p* < 0.001, *η*^2^ = 0.735] compared to non-painful pictures.

To investigate whether electrophysiological activity elicited by painful pictures correlated with both the pain intensity rating and the emotional reaction, the correlation between mean amplitudes of ERPs elicited by painful pictures and the measurement of subjective reports was calculated. However, no reliable correlations were found (all *p*-values were > 0.05).

### ERP data from the auditory experiment

**Figure 2:**
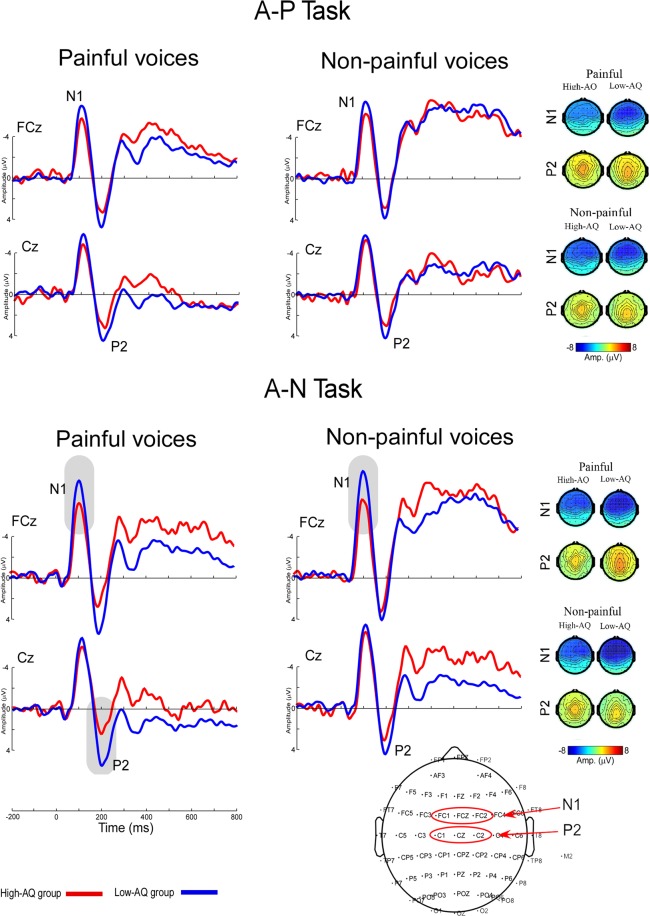
Cortical responses to painful and non-painful voices at FCz and Cz in the auditory experiment. Top panel: ERP waves and voltage scalp maps of the High-AQ group in response to the painful and non-painful voices in the Auditory A-P task. Bottom panel: ERP waves and voltage scalp maps of the High-AQ and Low-AQ groups in response to the painful and non-painful voices in the Auditory A-N task. Red lines illustrate averaged amplitudes of the High-AQ group. Blue lines illustrate averaged amplitudes of the Low-AQ group. The High-AQ group elicited smaller N1 waves than the Low-AQ group in the A-N task (*p* = 0.030, outlined in gray shaded area), whereas no difference was found between the two groups in the A-P task (*p* = 0.223). The High-AQ group elicited smaller P2 waves than the Low-AQ group in response to painful voices in the A-N task (*p* = 0.002, outlined in gray shaded area), whereas no difference was found between the two groups in response to painful voices in the A-P task, and non-painful voices in both of the A-P and A-N tasks (*p* > 0.05). Topographic plots at the bottom illustrate the electrodes considered in the analysis.

Averaged ERP waveforms and scalp topographies of each condition are shown in Figure 2. A full list of all statistical comparisons can be found in Table 4.

**Table 4:** Summary of statistical analysis of ERP data in the auditory experiment.

		N1			P2		
		*F*	*p*	*η* ^2^	*F*	*p*	*η* ^2^
Task	**amplitude**	**17.899**	**<0.001**	**0.390**	2.741	0.109	0.089
	**latency**	0.018	0.893	0.001	0.101	0.753	0.004
Pain	**amplitude**	2.237	0.146	0.074	**4.408**	**0.045**	**0.136**
	**latency**	3.431	0.075	0.109	0.366	0.550	0.013
Group	**amplitude**	3.147	0.087	0.101	**4.331**	**0.047**	**0.134**
	**latency**	0.641	0.430	0.022	0.166	0.687	0.006
Task × Group	**amplitude**	**5.112**	**0.032**	**0.154**	**7.693**	**0.010**	**0.216**
	**latency**	0.005	0.946	0.000	0.008	0.928	0.000
Pain × Group	**amplitude**	0.006	0.939	0.000	**5.389**	**0.028**	**0.161**
	**latency**	0.335	0.567	0.012	0.009	0.927	0.000
Task × Pain	**amplitude**	1.157	0.291	0.040	0.142	0.709	0.005
	**latency**	1.451	0.238	0.049	0.000	1.000	0.000
Task × Pain × Group	**amplitude**	1.389	0.248	0.047	**8.313**	**0.007**	**0.229**
	**latency**	**5.804**	**0.023**	**0.172**	1.183	0.286	0.041

Note: df:(1,28). The significant comparisons (*p* < 0.05) were shown in boldface.

#### ERPs amplitude

The N1 amplitudes were modulated by “task” [*F*(1,28) = 17.899, *p* < 0.001, *η*^2^ = 0.390] and the N1 amplitude was higher in the A-N task (−7.88 ± 0.55 μV) than in the A-P task (−6.93 ± 0.60 μV). The N1 amplitudes were also modulated by the interaction between “task” and “group” [*F*(1,28) = 5.112, *p* = 0.032, *η*^2^ = 0.154]. Post hoc two-way ANOVA showed that the High-AQ group (−6.63 ± 0.78 μV) elicited smaller N1 waves than the Low-AQ group (−9.14 ± 0.78 μV) in the A-N task [*F*(1,28) = 5.224, *p* = 0.030, *η*^2^ = 0.157]. No difference was found between both groups (High-AQ group: −6.18 ± 0.85 μV; Low-AQ group: −7.68 ± 0.85 μV) in the A-P task [*F*(1,28) = 1.552, *p* = 0.223, *η*^2^ = 0.053].

The P2 amplitude was modulated by “group” [*F*(1,28) = 4.331, *p* = 0.047, *η*^2^ = 0.134] and “pain” [*F*(1,28) = 4.408, *p* = 0.045, *η*^2^ = 0.136].The High-AQ group (2.95 ± 0.71 μV) showed smaller P2 amplitudes than the Low-AQ group (5.02 ± 0.70 μV). Painful vocal cues (4.23 ± 0.54 μV) elicited higher P2 amplitudes than non-painful vocal cues (3.75 ± 0.48 μV). Notably, P2 amplitudes were significantly modulated by the interaction among “task” and “group” [*F*(1,28) = 7.693, *p* = 0.010, *η*^2^ = 0.216], “pain” and “group” [*F*(1,28) = 5.389, *p* = 0.028, *η*^2^ = 0.161], as well as “task”, “pain”, and “group” [*F*(1,28) = 8.313, *p* = 0.007, *η*^2^ = 0.229]. To better understand these interactions, the High-AQ and Low-AQ groups were compared in all conditions: the High-AQ group (2.35 ± 2.77 μV) elicited smaller P2 waves than the Low-AQ group (5.88 ± 2.80 μV) in response to painful voices in the A-N task [*F*(1,28) = 12.031, *p* = 0.002, *η*^2^ = 0.301], which passed FDR correction. However, no difference was found between the two groups in response to (1) painful voices in the A-P task [*F*(1,28) = 2.015, *p* = 0.167, *η*^2^ = 0.067; High-AQ group: 3.50 ± 3.28 μV, Low-AQ group: 5.18 ± 3.22 μV], (2) non-painful voices in the A-P task [*F*(1,28) = 2.719, *p* = 0.110, *η*^2^ = 0.089; High-AQ group: 3.14 ± 2.71 μV, Low-AQ group: 4.68 ± 2.39 μV], and (3) non-painful voices in the A-N task [*F*(1,28) = 2.226, *p* = 0.147, *η*^2^ = 0.074; High-AQ group: 2.80 ± 3.07 μV, Low-AQ group: 4.35 ± 2.62 μV].

#### ERPs latency

N1 latencies were significantly modulated by the interaction between “task”, “pain”, and “group” [*F*(1,28) = 5.804, *p* = 0.023, *η*^2^ = 0.172]. To better understand this interaction, the High-AQ and Low-AQ groups were compared in all conditions; however, no difference between both groups was obtained after FDR correction: (1) painful voices in the A-P task [*F*(1,28) = 0.012, *p* = 0.913, *η*^2^ < 0.001; High-AQ group: 114.93 ± 3.59 ms, Low-AQ group: 111.20 ± 3.42 ms], (2) painful voices in the A-N task [*F*(1,28) = 1.543, *p* = 0.224, *η*^2^ = 0.052; High-AQ group: 112.53 ± 3.19 ms, Low-AQ group: 106.93 ± 3.19 ms], (3) non-painful voices in the A-P task [*F*(1,28) = 1.727, *p* = 0.200, *η*^2^ = 0.058; High-AQ group: 114.93 ± 3.59 ms, Low-AQ group: 108.28 ± 3.59 ms], and (4) non-painful voices in the A-N task [*F*(1,28) = 0.054, *p* = 0.818, *η*^2^ = 0.002; High-AQ group: 113.87 ± 3.24 ms, Low-AQ group: 112.80 ± 3.24 ms].

#### Subjective reports and their correlation with neural activity

Mean scores and standard deviations of subjective reports about the painful and non-painful voices are shown in Table 1. A full list of all statistical comparisons can be found in Table 2.

Participants judged painful voices with higher pain intensities [*F*(1,28) = 289.627, *p* < 0.001, *η*^2^ = 0.912] and more negative emotional reactions [*F*(1,28) = 31.698, *p* < 0.001, *η*^2^ = 0.531] than non-painful voices. To investigate whether the electrophysiological activities that were elicited by the voices were correlated with pain intensity ratings and emotional reactions, the correlation between the mean amplitudes of ERP components and the measurements obtained from subjective reports was calculated.

For High-AQ participants, the P2 amplitudes related to painful voices in the auditory A-P task were significantly correlated with the pain intensity ratings [*r*(15) = 0.646, *p* = 0.009] but were not correlated with emotional reactions [*r*(15) = 0.075, *p* = 0.789]. However, for Low-AQ participants, the mean P2 amplitudes elicited by painful voices were neither correlated with pain intensity ratings, nor with emotional reactions (all *p*-values were >0.05). No other reliable correlation was found between subjective reports and ERP peak amplitudes.

### ERPs data from visual and auditory experiments combined

For N1 and P2 ERP components in the visual and auditory experiments, peak amplitudes and latencies were compared via mixed model ANOVA using the between-participants factor “group” and the within-participants factors “modality”, “task”, and “pain”. A full list of all statistical comparisons can be found in Table 5.

**Table 5:** Summary of statistical analysis of N1 and P2 components for visual and auditory experiments combined.

Experiment	N1 amplitude			N1 latency			P2 amplitude			P2 latency		
	*F*	*p*	*η* ^2^	*F*	*p*	*η* ^2^	*F*	*p*	*η* ^2^	*F*	*p*	*η* ^2^
Task	**21**.**918**	<**0**.**001**	**0**.**439**	0.132	0.719	0.005	**15**.**662**	<**0**.**001**	**0**.**359**	0.021	0.886	0.001
Pain	1.736	0.198	0.058	0.071	0.791	0.003	**5**.**491**	**0**.**026**	**0**.**164**	0.168	0.685	0.006
Group	1.108	0.302	0.038	0.589	0.449	0.021	**6**.**033**	**0**.**021**	**0**.**177**	0.125	0.726	0.004
Modality	3.598	0.068	0.114	**48**.**577**	<**0**.**001**	**0**.**634**	**16**.**775**	<**0**.**001**	**0**.**375**	**4**.**683**	**0**.**039**	**0**.**143**
Task × Group	2.179	0.151	0.072	0.001	0.981	<0.001	1.284	0.267	0.044	0.001	0.971	<0.001
Pain × Group	1.185	0.286	0.041	0.071	0.791	0.003	0.472	0.498	0.017	0.138	0.713	0.005
Task × Pain	2.127	0.156	0.071	0.006	0.938	<0.001	0.572	0.456	0.020	0.031	0.861	0.001
Modality × Task	1.085	0.307	0.037	0.036	0.850	0.001	**6**.**206**	**0**.**019**	**0**.**181**	0.172	0.682	0.006
Modality × Group	2.868	0.101	0.093	0.022	0.884	0.001	0.024	0.877	0.001	1.044	0.316	0.036
Modality × Task × Group	2.129	0.156	0.071	0.019	0.893	0.001	2.252	0.145	0.074	0.015	0.902	0.001
Modality × Pain × Group	1.159	0.291	0.040	0.064	0.802	0.002	**7**.**500**	**0**.**011**	**0**.**211**	0.056	0.814	0.002
Pain × Group × Task	0.188	0.668	0.007	3.301	0.080	0.105	**4**.**538**	**0**.**042**	**0**.**139**	0.244	0.626	0.009
Modality × Task × Pain	0.091	0.765	0.003	1.264	0.270	0.043	0.153	0.699	0.005	0.023	0.879	0.001
Modality × Pain × Group × Task	1.268	0.270	0.043	0.272	0.606	0.010	0.949	0.338	0.033	1.218	0.279	0.042

Note: df:(1,28). The significant (*p* < 0.05) comparisons were shown in boldface.

For ERP components of the visual and auditory experiments, both N1 and P2 amplitudes were significantly modulated by “task” [N1: *F*(1,28) = 21.918, *p* < 0.001, *η*^2^ = 0.439; P2: *F*(1,28) = 15.662, *p* < 0.001, *η*^2^ = 0.359]. The N1 amplitudes were greater in the A-N task (−7.07 ± 0.34 μV) than in the A-P task (−6.32 ± 0.38 μV) and the P2 amplitudes were smaller in the A-N task (2.22 ± 0.39 μV) than in the A-P task (2.93 ± 0.43 μV), indicating a more negative deflection of ERP waves in the A-N task than in the A-P task.

The P2 amplitudes were significantly modulated by “group” [*F*(1,28) = 6.033, *p* = 0.021, *η*^2^ = 0.177], “pain” [*F*(1,28) = 6.926, *p* = 0.014, *η*^2^ = 0.198], and “modality” [*F*(1,28) = 16.775, *p* < 0.001, *η*^2^ = 0.375]. The High-AQ group (1.59 ± 0.57 μV) showed smaller P2 amplitudes than the Low-AQ group (3.56 ± 0.57 μV). Painful stimuli (2.77 ± 0.44 μV) elicited higher P2 amplitudes than non-painful stimuli (2.38 ± 0.37 μV). P2 amplitudes in the auditory modality (3.99 ± 0.50 μV) were larger than in the visual modality (1.17 ± 0.56 μV). P2 amplitudes were modulated by the interaction between “task” and “modality” [*F*(1,28) = 6.206, *p* = 0.019, *η*^2^ = 0.181]. P2 amplitudes in the A-P task (1.74 ± 0.60 μV) were larger than that in the A-N task (0.60 ± 0.55 μV) in the visual modality [*F*(1,28) = 13.498, *p* = 0.001, *η*^2^ = 0.325], whereas no significant difference was found between the A-P task (4.12 ± 0.52 μV) and the A-N task (3.85 ± 0.49 μV) in the auditory modality [*F*(1,28) = 2.741, *p* = 0.109, *η*^2^ = 0.089]. Notably, P2 amplitudes were modulated by the interaction among “task”, “pain”, and “group” [*F*(1,28) = 4.538, *p* = 0.042, *η*^2^ = 0.139]. The results of post hoc two-way ANOVA showed that High-AQ participants elicited smaller P2 amplitudes than Low-AQ participants in response to painful stimuli [*F*(1,28) = 9.515, *p* = 0.005, *η*^2^ = 0.254; High-AQ group: 1.17 ± 0.60 μV, Low-AQ group: 3.77 ± 0.60 μV] and non-painful stimuli [*F*(1,28) = 4.744, *p* = 0.038, *η*^2^ = 0.145; High-AQ group: 1.10 ± 0.56 μV, Low-AQ group: 2.84 ± 0.56 μV] in the A-N task and non-painful stimuli in the A-P task [*F*(1,28) = 6.961, *p* = 0.013, *η*^2^ = 0.199; High-AQ group: 1.81 ± 0.53 μV, Low-AQ group: 3.78 ± 0.53 μV]. However, no difference was found between groups in their responses to the painful stimuli in the A-P task [*F*(1,28) = 2.461, *p* = 0.128, *η*^2^ = 0.081; High-AQ group: 2.29 ± 0.70 μV, Low-AQ group: 3.85 ± 0.70 μV].

N1 latencies were modulated by “modality” [*F*(1,28) = 48.577, *p* < 0.001, *η*^2^ = 0.634], and N1 latencies in the auditory modality (111.40 ± 2.00 ms) were shorter than in the visual modality (134.97 ± 3.65 ms).

## Discussion

The present study investigated the influence of autistic traits on top-down attention-induced modulation regarding empathy for pain in both visual and auditory modalities. In line with previous studies^3,4^, top-down attention for the pain of others was manipulated by instructing the participants to either pay attention to pain cues in the stimuli presented in the A-P task, or to pay attention to non-pain cues in the stimuli presented in the A-N task. Electrophysiological results showed that the influence of autistic traits on attention manipulation of empathy for pain was only exhibited in the auditory modality, i.e., High-AQ participants exhibited suppressed N1 and P2 waves in response to others’ painful voices in comparison to Low-AQ participants in the auditory A-N task. No significant difference was found between groups in the auditory A-P task, or in the visual A-P/A-N tasks. These results indicated that High-AQ individuals exhibited a decreased implicit neural response to others’ auditory pain signals. This insight can guide interventions to improve the overall empathic competence of ASD individuals, e.g., by combining explicit and implicit instructions to improve empathy for pain in the auditory modality.

The present study adopted an empathy for pain task in both visual and auditory modalities. Analyses of the main effects and interactions of “modality” indicated that participants judged less accurately and effectively when using their auditory modality than when using their visual modality, especially with regard to non-painful stimuli in A-N tasks. In addition, P2 amplitudes in the auditory modality were larger than in the visual modality, suggesting that participants should utilise more mental recourse in the auditory modality than visual modality in these components. These results suggest that tasks that involve the auditory modality may be more difficult than those involving the visual modality.

Consistent with previous studies that reporter longer RTs in A-P tasks than in A-N tasks^3^, in the present study, both RTs and IES were also significantly modulated by “task”. In addition, participants responded less accurately to the A-N task than to the A-P task, suggesting the A-N task was more difficult than the A-P task. Importantly, the present study showed that the High-AQ group responded slower than the Low-AQ group. This was in line with a previous study, which showed that individuals with ASD and autistic traits required more time to make decisions than the control group^16^. In addition, ACCs, RTs, IESs, P3, and LPC amplitudes in the visual experiment, as well as P2 amplitude in the auditory experiment were significantly modulated by “pain”: participants in both groups responded significantly more accurately and faster, as well as elicited higher ERP amplitudes to painful stimuli than to non-painful stimuli. This indicates a processing bias towards others’ pain, as reflected by both behavioural and neural response.

For the painful pictures, the P3 and LPC amplitudes were significantly modulated by “task”, i.e., the A-P task elicited larger amplitudes for painful pictures than the A-N task, which was consistent with the previous suggestion that late empathic responses to others’ pain were modulated by top-down attention to the pain cues^3^. P3 and LPC components over the posterior parietal area have been linked to stimulus evaluation processes^17,18^. Hence, the patterns for P3 and LPC components might reflect a greater use of cognitive resources in response to painful pictures in the A-P task than in the A-N task. Moreover, P3 latencies were consistent with behavioural data showing that High-AQ participants responded much slower than Low-AQ participants. This result indicates differences in stimulus evaluation processes between Low-AQ and High-AQ participants, and High-AQ participants required more time to evaluate others’ pain than Low-AQ participants.

In the auditory experiment, High-AQ participants responded much slower than Low-AQ participants, confirming the between-group difference of decision-making biases, i.e., individuals with autistic traits may be reluctant to make quick decisions and are more prone to choosing a more deliberative process than control groups^16,19^. The electrophysiological results showed that N1 amplitudes were higher in the auditory A-N task than in the auditory A-P task, as greater attention resource allocation^20,21^ was required in the auditory A-N task.

Importantly, both N1 and P2 amplitudes, elicited by vocal stimuli, were significantly modulated by interactions among “group” and “task” in response to others’ painful voices. In comparison to the Low-AQ group, the High-AQ group showed suppressed N1 and P2 amplitudes in response to painful voices when they were instructed to focus on non-pain cues (auditory A-N task). However, when they were instructed to focus on pain cues (auditory A-P task), both N1 and P2 amplitudes displayed similar brain responses to painful and non-painful voices. Previous studies have shown that a higher level of attention to upcoming external auditory stimuli induces a larger N1 amplitude when attention was directed toward the stimulation^20,21^. This implies that High-AQ individuals paid less attention to others’ pain than Low-AQ individuals when they were instructed to judge the gender of the voices (auditory A-N task), in comparison to when they were instructed to judge their level of pain (auditory A-P task). According to the perceptual model of attention^22,23^, an alternative explanation would be that the decreased perceptual load resulted in a suppressed N1 response. Consequently, the perceptual load in the A-N task (judging others’ gender) was lower for High-AQ participants than for Low-AQ participants. This speculation is supported by previous studies reporting that individuals with autistic traits exhibited greater competence in systematic tasks^24^.

Previous findings have also shown that the P2 response is relevant to the emotional quality of the presented external sensory stimulation^25^. High-AQ individuals showed a smaller P2 amplitude than Low-AQ individuals (i.e., less emotional arousal) to the pain of others when they were instructed to judge others’ gender (auditory A-N task) while ignoring the pain cues in their voices. In addition, High-AQ participants showed a positive correlation between the P2 amplitude and subjective pain intensity ratings in the auditory A-P task, i.e., a higher P2 amplitude evoked by the stimulus, leading to stronger pain reports. These results suggest suppressed implicit empathic processing in individuals with autistic traits^13,26^.

In summary, this study found that High-AQ participants’ responses to others’ vocal pain were modulated by top-down attention, while such an effect was not found for the visual modality. This is consistent with previous reports about discrete responses of High-AQ individuals to others’ pain, or emotion recognition, between visual and auditory modalities^15,27,28^. Indeed, this finding not only expands our understanding about the influence of autistic traits on individual empathic responses, but also suggests that interventions with a sole focus on visual appraisals of others’ emotional information may not be effective to improve the overall empathic competence of ASD individuals.

Despite these possible implications, several limitations of the present study should also be noted. First, this study only used pictures of static physical pain and single painful voices to evaluate the empathic abilities of participants. Further investigations should use dynamic videos to evaluate the individual empathic process. Second, although the influence of autistic traits on top-down attention modulation on empathic responses was assessed under experimental settings, whether and how these responses relate to real-world empathy for pain requires further investigation. Third, although it has been shown that ASD individuals and healthy controls did not distinctively process auditory stimuli at different frequencies^29^, possible influences caused by different frequency bands of non-painful and painful voices cannot be completely eliminated (such as lower frequency for neutral voices and higher frequency for painful voices).

## Conclusion

Autistic traits are distributed across the population, and individuals with ASD score at the extreme end of this distribution^7^. To investigate the association between autistic traits and the empathy for pain in TD adults, this study employed the AQ to quantify autistic traits in healthy adults. This study investigated whether empathic responses to others’ pain by Low-AQ and High-AQ individuals were modulated differently by top-down attention, in both visual and auditory modalities. Distinctive top-down attention modulation of responses to others’ pain was identified between Low-AQ and High-AQ participants in the auditory modality but not in the visual modality. Relative to Low-AQ individuals, painful vocal stimuli elicited suppressed N1 and P2 amplitudes when High-AQ individuals were instructed to pay attention to non-pain cues, whereas no such difference was found when they were instructed to pay attention to the pain cues. These results suggest that the top-down attention modulation of cortical empathic responses to others’ audible pain is influenced by autistic traits.

## Methods

### Participants

A total of 1,231 university students at the Chongqing Normal University, aged 18–23 (mean = 19.7 years, SD = 2.2 years) were recruited to complete the Mandarin Version^30^ of the AQ questionnaire^6^, which was used to estimate their autistic traits. Then, 15 participants (7 females), randomly selected from those displaying the 10% highest AQ scores, and 15 participants (7 females), randomly selected from those displaying the 10% lowest AQ scores, were identified as High-AQ group and Low-AQ group, respectively^15^. Their ages and AQ scores are summarized in Table 6. These participants were further recruited to participate in the electroencephalography (EEG) recording experiment.

**Table 6:** Ages and AQ scores of High-AQ and Low-AQ participants in the study.

	Age			AQ Score		
	Mean (SD)	*t* (df = 28)	*p*	Mean (SD)	*t* (df = 28)	*p*
High-AQ	19.12 (1.41)	−0.661	0.514	28.40 (1.30)	31.195	<0.001
Low-AQ	19.47 (1.35)			10.07 (1.87)		

Note. AQ = Autism Spectrum Quotient. *p*-values and t values were obtained from independent samples *t*-tests performed on ages and AQ scores between High-AQ and Low-AQ participant.

All participants gave their free and informed consent to the study before the experiment in accordance with the Declaration of Helsinki and all procedures were approved by the Chongqing Normal University research ethics committee. The procedures were performed in accordance with current ethical guidelines and regulations.

### Stimuli

#### Visual stimuli

**Figure 3:**
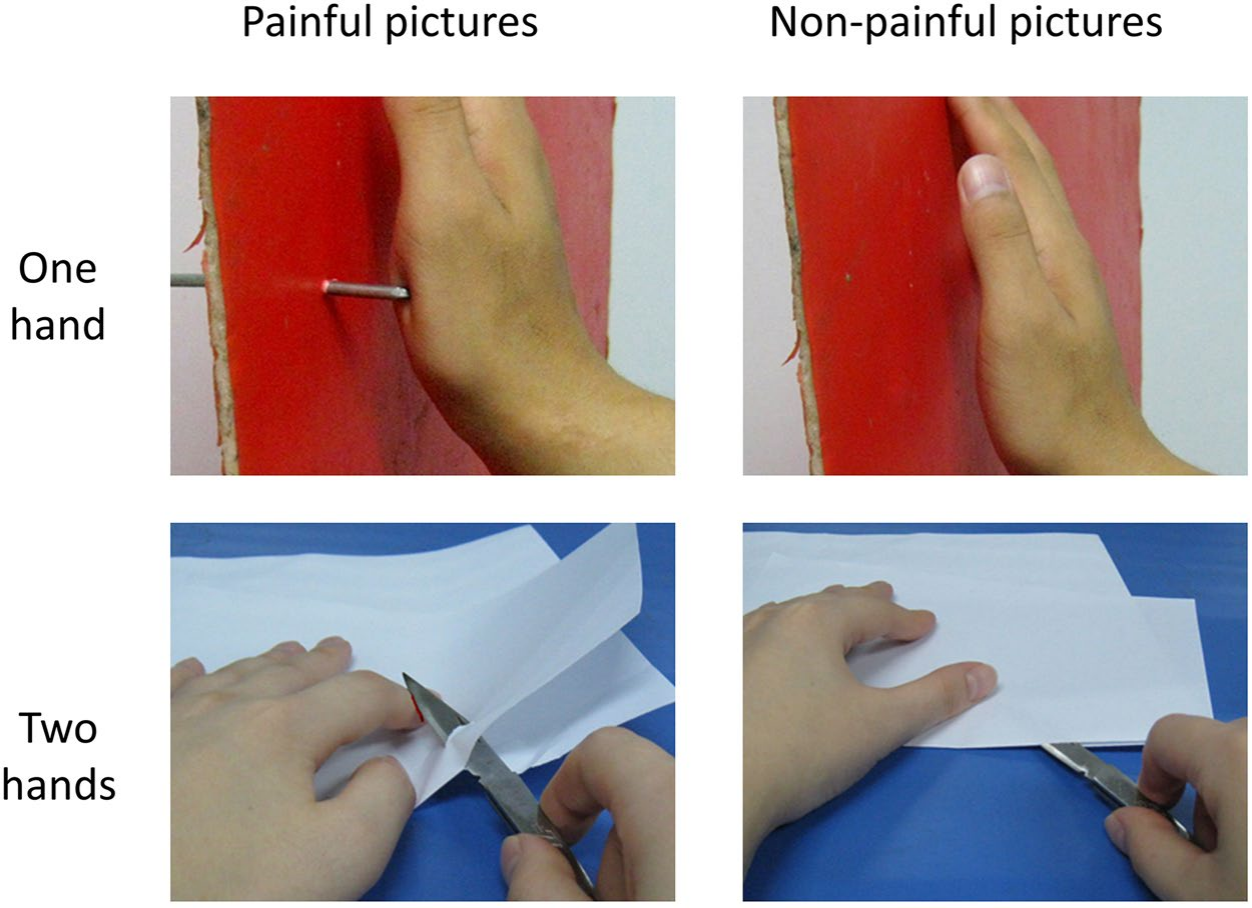
Examples of painful (left panel) and non-painful pictures (right panel). Examples of pictures with one hand (top panel) and two hands (bottom panel).

A total of 70 pictures (consisting of 35 painful and 35 non-painful pictures) were selected from a picture database that was previously validated and used in published studies^15,31,32^. Each picture depicted a familiar event that might occur in everyday life. Each painful picture depicted a model with either one hand or both hands involved in a painful situation, e.g., cutting oneself with a knife. Non-painful pictures were of a similar nature but did not display any painful components. Luminance, contrast, and colour were matched between both groups of pictures. Thirty-six pictures showed one hand (17 painful and 19 non-painful scenes) and 34 pictures showed two hands (18 painful and 16 non-painful scenes). Two pairs of example pictures are shown in Figure 3. All pictures were flipped horizontally using Adobe Photoshop CC 2018. The visual stimuli used for the visual experiment consisted of 140 digital colour pictures with 70 original pictures and 70 flipped pictures.

#### Auditory stimuli

A total of 20 audio recordings of interjections (/α/), that were spoken with either a painful (10 recordings) or neutral (10 recordings) prosody, were selected from the Montreal Affective Voices database^33^. These samples were recorded by 10 actors (five male and five female). All audio clips were edited to be 700 ms long^25^, with a mean intensity of 70 dB^34^.

### EEG recording

EEG data were recorded from 64 scalp sites using tin electrodes mounted on an elastic cap (Neuroscan 4.3, Neurosoft, Inc., Sterling, VA, USA). The electrode at the right mastoid was used as recording reference and the electrode on the medial frontal aspect was used as ground electrode. Vertical electrooculograms (EOGs) were recorded both supra- and infra-orbitally at the left eye. Horizontal EOGs were recorded as left versus right orbital rim. EEG and EOG activities were amplified with a DC ~100 Hz bandpass and were continuously sampled at 500 Hz. All electrode impedances remained below 5 kΩ.

### Procedure

Participants were seated in a quiet room with an ambient temperature of about 20 °C. The order of experimental modalities was counterbalanced between participants. For both experiments, test items were presented at random order. Stimulus presentation was controlled using the E-Prime (3.0) program.

#### Visual experiment

The visual experiment consisted of two sessions involving different tasks: the attention to pain cue tasks (visual A-P task) and the attention to non-pain cue tasks (visual A-N task). For the visual A-P task, participants were instructed to judge whether the depicted scene was painful or non-painful, while for the visual A-N task, participants were instructed to count the number of hands in the depicted situations, responding by pressing a key using only their right hand. The order of experimental tasks (visual A-P and visual A-N) was counterbalanced between participants. Prior to each task, a training session was conducted during which each participant could familiarize with the experimental procedures. This training session consisted of eight trials, where two painful pictures and two non-painful pictures were selected from the picture database^15,31,32^. These specific pictures were excluded from the main experiment.

**Figure 4:**
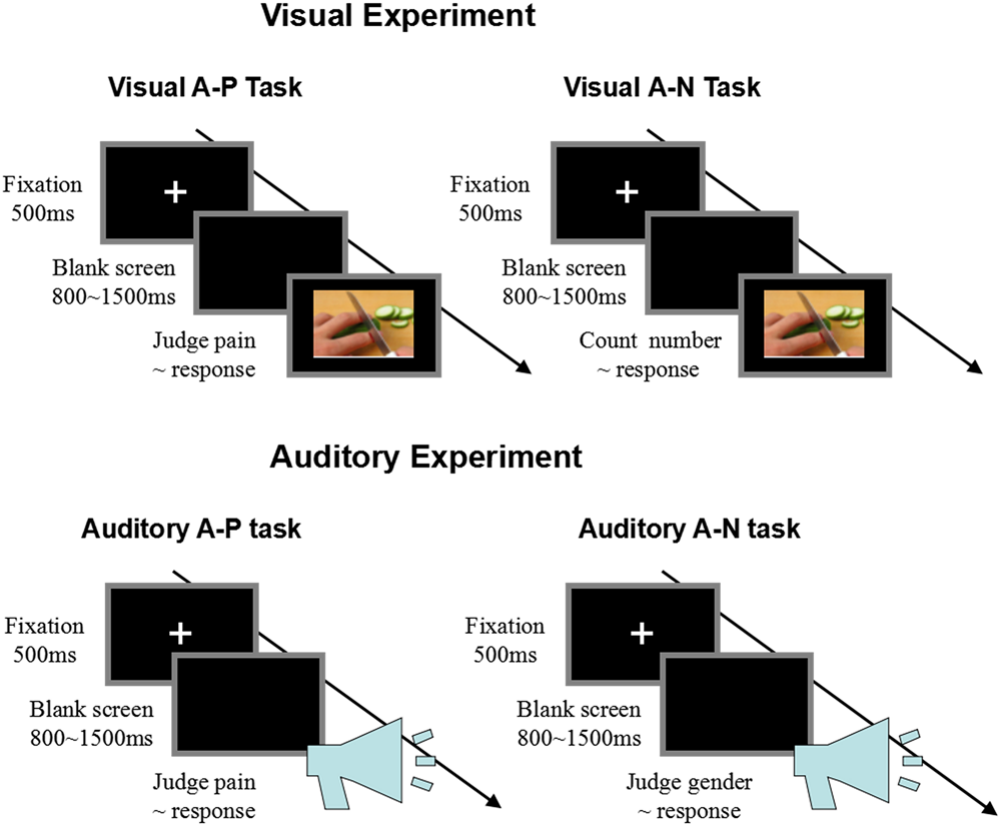
Flowchart describing the experimental designs of visual and auditory experiments. Top left column: Procedure of Visual A-P tasks. Top right column: Procedure of Visual A-N tasks. Bottom left column: Procedure of Auditory A-P tasks. Bottom right column: Procedure of Auditory A-N tasks.

An example trial of the visual A-P task is displayed within the top-left column of Figure 4. At the start of the trial, a fixation cross was presented on a black screen for a duration of 500 ms. 800–1,500 ms later, a picture was presented, and the participants were instructed to respond as accurately and quickly as possible by pressing a key (either “1” or “2”) to judge whether the picture was painful or non-painful. The picture disappeared from the screen as soon as the participant had provided their response. The key-pressing was counterbalanced across participants to control for order effects. The visual A-P task comprised of two blocks with 70 trials per block and an inter-trial interval of 1,000 ms. Each picture was presented only once for this task.

The experimental procedures of the visual A-N task were identical to the procedures of the visual A-P task, with the exception that participants were instructed to respond as accurately and quickly as possible, by pressing the respective key (“1” or “2”) to report the number of hands they saw in the pictures (see the top-right column in Figure 4).

#### Auditory experiment

The auditory experiment consisted of two sessions with different tasks: auditory A-P tasks, in which participants were instructed to judge whether the voices were painful or non-painful, and auditory A-N tasks in which participants were instructed to judge the gender of the speakers as either female or male. The response required the pressing of a key using only their right hand. The order of the experimental tasks (auditory A-P and auditory A-N) was counterbalanced. Prior to each task, a training session was conducted for each participant. This training session consisted of eight experimental trials which presented two painful voices (one of each gender) and two non-painful voices (also one of each gender), each lasting 1,000 ms. It should be noted that the duration of the audible voices differed between the training session (1,000 ms) and the main experimental session (700 ms). This longer presentation for training purposes was applied to aid the process of familiarizing participants with the experimental procedures. The particular samples used in the training session were not reused in the main experiment.

At the start of the auditory A-P trial (see the bottom-left column of Figure 4), a fixation cross was presented on a black screen for a duration of 500 ms. After the black screen lasting for 800–1,500 ms, 700 ms of a vocal recording was presented through earphones. Participants were instructed to respond as accurately and quickly as possible, by pressing a specific key (either “1” or “2”) to judge whether the voice was painful or non-painful. The key-pressing was counterbalanced across participants to control for order effects. The auditory A-P task comprised two blocks, with 70 trials in each block (140 trials in total, in pseudorandom order), and an inter-trial interval of 1,800–2,500 ms.

For auditory A-N tasks (see the bottom-right column of Figure 4), experimental procedures were identical to the procedures for auditory A-P tasks, except that participants were instructed to respond as accurately and quickly as possible, by pressing a key (“1” or “2”) to judge the gender of the speaker as female or male, respectively, judging from their voice alone.

#### Measurement of subjective reports

After the EEG recording session, participants were asked to rate the intensity of others’ pain in pictures and audio recordings based on a 9-point pain intensity scale (1 = no sensation, 4 = pain threshold, 9 = most intense pain imaginable). Participants were furthermore required to evaluate their subjective emotional reactions to these visual and auditory stimuli, again using a 9-point emotion scale (1 = extremely happy, 5 = neutral, 9 = extremely unhappy).

### Data analysis

#### Behavioural data

Data analyses were performed on both procedures. First, the recorded ACCs and RTs for pictures (visual experiment) and voices (auditory experiment) were compared via four-way repeated-measures analyses of variance (ANOVA), using three within-participant factors of “modality” (visual vs. auditory), “pain” (painful vs. non-painful), and “task” (A-P vs. A-N), as well as the between-participants factor of “group” (High-AQ vs. Low-AQ). If the interaction effect was significant, post hoc three-way repeated measures ANOVA was performed for Low-AQ and High-AQ groups, with within-participants factors “modality”, “task”, and “pain”. Second, ACCs and RTs were combined into a single score (the IES) to obtain a general performance index that discounts possible criterion shifts or speed-accuracy-trade-off effects^35^. As an adjusted RT measure, IES is derived by dividing RT by its corresponding percentage accuracy. It adjusts the RT performance for sacrifices in accuracy, which might have been made in favour of speed. RTs with high ACCs indicate smaller IESs (i.e., are more efficient) than the same RTs achieved at the cost of more errors^35^. The data were analysed using SPSS 15 software.

#### EEG data

EEG data were pre-processed and analysed via MATLAB 7.0 (MathWorks, USA) using the EEGLAB toolbox^36^. EEG signals were passed through an off-line 0.001-30 Hz band-pass filter. Time windows of 200 ms before and 1,000 ms after the onset of stimuli were extracted from the continuous EEG and the extracted window was baseline-corrected by the 200 ms time interval prior to stimuli onset. The epoched EEGs were inspected and trials that were contaminated by gross movements were removed. EOG artefacts were corrected via an independent component analysis (ICA) algorithm^37^. Epochs with amplitude values exceeding ±60 μV at any electrode were excluded from the presented average. Excluded epochs constituted 6 ± 2.4% of the total number of epochs.

After confirming voltage scalp maps in both single-participant and group-level ERP waveforms and previously reported sites^3,38,39^, dominant ERP components of the visual experiment were extracted from the following electrode sites: N1 (FCz, FC1, and FC2), P2 (Cz, C1, and C2), N2 (Fz, F1, and F2), P3 (Pz, P1, and P2), and LPC (Pz, P1, and P2). Mean latencies and amplitudes of these electrode sites were measured at electrodes that displayed maximal responses. In addition, dominant ERP components of the auditory experiment were extracted from electrode sites corresponding to the voltage scalp maps and previously reported sites^25,40,41^. For example, latencies and amplitudes of N1 (FCz, FC1, and FC2) and P2 (Cz, C1, and C2) were measured at electrodes that displayed maximal responses.

ERP data analyses were performed using two procedures: First, for N1 and P2, ERP components of both the visual and auditory experiments, peak amplitudes, and latencies were compared via mixed model ANOVA using the between-participants factor “group” and the within-participants factors “modality”, “task”, and “pain”. Second, peak amplitudes and latencies were analysed independently for each experiment. Peak latencies and amplitudes were compared via mixed model ANOVA using the between-participants factor “group” and the within-participants factors “task” and “pain”. The degrees of freedom for F-ratios were corrected according to the Greenhouse-Geisser method. If significant, post hoc ANOVA with factor “group” was performed for each condition. To account for the multiple comparison problem, the *p* values were corrected using a false discovery rate (FDR) procedure^42^.

### Ethics approval and consent to participate

This research was approved by the Chongqing Normal University research ethics committee. All participants had signed informed consent after being given a complete description of the study. The ethics committee approved this consent procedure.

## Data Availability

Supplementary data associated with this article can be found in the online version at https://pan.baidu.com/s/17Q18qJ1NIWy4VqB4mHELnw (Extraction code: yhy9).

## References

[1] Danziger N, Prkachin KM, Willer JC (2006). Is pain the price of empathy? The perception of others’ pain in patients with congenital insensitivity to pain. Brain.

[2] Decety J, Jackson PL (2004). The functional architecture of human empathy. Behav. Cogn. Neurosci. Rev..

[3] Fan Y, Han SH (2008). Temporal dynamic of neural mechanisms involved in empathy for pain: An event-related brain potential study. Neuropsychologia.

[4] Gu XS, Han SH (2007). Attention and reality constraints on the neural processes of empathy for pain. Neuroimage.

[5] Association, A. P. *Diagnostic and Statistical Manual of Mental Disorders (DSM-5®) (5th ed*.*)*. (American Psychiatric Pub, 2013).

[6] Baron-Cohen S, Wheelwright S, Skinner R, Martin J, Clubley E (2001). The autism-spectrum quotient (AQ): Evidence from asperger syndrome/high-functioning autism, malesand females, scientists and mathematicians. J. Autism Dev. Disord..

[7] Gökçen E, Petrides KV, Hudry K, Frederickson N, Smillie LD (2014). Sub-threshold autism traits: The role of trait emotional intelligence and cognitive flexibility. Br. J. Psychol..

[8] Poljac E, Poljac E, Wagemans J (2013). Reduced accuracy and sensitivity in the perception of emotional facial expressions in individuals with high autism spectrum traits. Autism.

[9] Halliday DW, MacDonald SW, Sherf SK, Tanaka JW (2014). A Reciprocal Model of Face Recognition and Autistic Traits: Evidence from an Individual Differences Perspective. PLoS One.

[10] Baron-Cohen S, Wheelwright S (2004). The Empathy Quotient: An Investigation of Adults with Asperger Syndrome or High Functioning Autism, and Normal Sex Differences. J. Autism Dev. Disord..

[11] Actis-Grosso, R., Bossi, F. & Ricciardelli, P. Emotion recognition through static faces and moving bodies: a comparison between typically developed adults and individuals with high level of autistic traits. *Front*. *Psychol*. **6** (2015). 10.3389/fpsyg.2015.01570 PMC4615932 26557101

[12] Begeer S, Koot HM, Rieffe C, Terwogt MM, Stegge H (2008). Emotional competence in children with autism: Diagnostic criteria and empirical evidence. Dev. Rev..

[13] Kliemann D, Rosenblau G, Bölte S, Heekeren HR, Dziobek I (2013). Face puzzle—two new video-based tasks for measuring explicit and implicit aspects of facial emotion recognition. Front. Psychol..

[14] Greenwald AG, Banaji MR (1995). Implicit social cognition: attitudes, self-esteem, and stereotypes. Psychol. Rev..

[15] Meng J, Li Z, Shen L (2017). Responses to others’ pain in adults with autistic traits: The influence of gender and stimuli modality. PLoS One.

[16] Farmer GD, Baron-Cohen S, Skylark WJ (2017). People With Autism Spectrum Conditions Make More Consistent Decisions. Psychol. Sci..

[17] McCarthy G, Donchin E (1981). A metric for thought - a comparison of P300 latency and reaction time. Science.

[18] Olofsson JK, Nordin S, Sequeira H, Polich J (2008). Affective picture processing: An integrative review of ERP findings. Biol Psychol.

[19] Luke L, Clare IC, Ring H, Redley M, Watson P (2012). Decision-making difficulties experienced by adults with autism spectrum conditions. Autism the International Journal of Research & Practice.

[20] Choi I, Wang L, Bharadwaj H, Shinncunningham B (2014). Individual differences in attentional modulation of cortical responses correlate with selective attention performance. Hearing Res.

[21] Slagter HA, Prinssen S, Reteig LC, Mazaheri A (2016). Facilitation and inhibition in attention: Functional dissociation of pre-stimulus alpha activity, P1, and N1 components. Neuroimage.

[22] Rorden C, Guerrini C, Swainson R, Lazzeri M, Baylis GC (2008). Event related potentials reveal that increasing perceptual load leads to increased responses for target stimuli and decreased responses for irrelevant stimuli. Front. Hum. Neurosci..

[23] Lavie N (2005). Distracted and confused?: Selective attention under load. Trends Cogn Sci.

[24] Wheelwright S (2006). Predicting autism spectrum quotient (AQ) from the systemizing quotient-revised (SQ-R) and empathy quotient (EQ). Brain Res.

[25] Yeh P, Geangu E, Reid V (2016). Coherent emotional perception from body expressions and the voice. Neuropsychologia.

[26] Kana RK, Patriquin MA, Black BS, Channell MM, Wicker B (2016). Altered Medial Frontal and Superior Temporal Response to Implicit Processing of Emotions in Autism. Autism Research Official Journal of the International Society for Autism Research.

[27] Lerner MD, McPartland JC, Morris JP (2013). Multimodal emotion processing in autism spectrum disorders: An event-related potential study. Dev. Cogn. Neurosci..

[28] Jones CRG (2011). A multimodal approach to emotion recognition ability in autism spectrum disorders. Journal of Child Psychology and Psychiatry.

[29] Čeponienė R (2003). Speech-sound-selective auditory impairment in children with autism: They can perceive but do not attend. Proc. Natl. Acad. Sci. USA.

[30] Liu M-J (2008). Screening Adults for Asperger Syndrome and High-Functioning Autism by Using the Autism-Spectrum Quotient (AQ) (Mandarin Version). Bulletin of Special Education.

[31] Meng, J. *et al*. Pain perception in the self and observation of others: An ERP investigation. *Neuroimage*, 164–173 (2013). 10.1016/j.neuroimage.2013.01.024 23376492

[32] Meng J (2012). Emotional primes modulate the responses to others’ pain: An ERP study. Exp Brain Res.

[33] Belin P, Fillion-Bilodeau S, Gosselin F (2008). The Montreal Affective Voices: A validated set of nonverbal affect bursts for research on auditory affective processing. Behav. Res. Methods.

[34] Pinheiro AP, Barros C, Pedrosa J (2016). Salience in a social landscape: electrophysiological effects of task-irrelevant and infrequent vocal change. Soc. Cogn. Affect. Neurosci..

[35] Bruyer R, Brysbaert M (2011). Combining speed and accuracy in cognitive psychology: Is the inverse efficiency score (IES) a better dependent variable than the mean reaction time (RT) and the percentage of errors (PE)?. Psychol. Belg..

[36] Delorme A, Makeig S (2004). EEGLAB: an open source toolbox for analysis of single-trial EEG dynamics including independent component analysis. J. Neurosci. Methods.

[37] Jung TP (2001). Analysis and visualization of single-trial event-related potentials. Hum Brain Mapp.

[38] Decety J, Yang CY, Cheng YW (2010). Physicians down-regulate their pain empathy response: An event-related brain potential study. Neuroimage.

[39] Fan YT, Chen CY, Chen SC, Decety J, Cheng YW (2014). Empathic arousal and social understanding in individuals with autism: Evidence from fMRI and ERP measurements. Soc. Cogn. Affect. Neurosci..

[40] Donkers FCL (2015). Attenuated Auditory Event-Related Potentials and Associations with Atypical Sensory Response Patterns in Children with Autism. J. Autism Dev. Disord..

[41] Jessen S, Kotz SA (2011). The temporal dynamics of processing emotions from vocal, facial, and bodily expressions. Neuroimage.

[42] Benjamini Y, Hochberg Y (1995). Controlling The False Discovery Rate - A Practical And Powerful Approach To Multiple Testing. Journal of the Royal Statistical Society.

